# Ghana’s Ensure Mothers and Babies Regular Access to Care (EMBRACE) program: study protocol for a cluster randomized controlled trial

**DOI:** 10.1186/s13063-014-0539-3

**Published:** 2015-01-27

**Authors:** Kimiyo Kikuchi, Evelyn Ansah, Sumiyo Okawa, Akira Shibanuma, Margaret Gyapong, Seth Owusu-Agyei, Abraham Oduro, Gloria Quansah-Asare, Abraham Hodgson, Masamine Jimba, for the Ghana EMBRACE Implementation Research Project

**Affiliations:** Department of Community and Global Health, Graduate School of Medicine, The University of Tokyo, 7-3-1 Hongo, Bunkyo-ku, Tokyo, 113-0033 Japan; Research and Development Division, Ghana Health Service, MB 190 Accra, Ghana; Dodowa Health Research Centre, PO Box DD1, Dodowa, Greater Accra Ghana; Kintampo Health Research Centre, PO Box 200, Kintampo, Brong-Ahafo Ghana; Navrongo Health Research Centre, PO Box 114, Navrongo, Upper East Ghana; Ghana Health Service, Accra, Ghana

**Keywords:** Continuum of Care, Maternal, Newborn, and Child Health Outcomes, Ghana

## Abstract

**Background:**

The United Nations’ Millennium Development Goals call for improving maternal and child health status. Their progress, however, has been minimal and uneven across countries. The continuum of care is a key to strengthening maternal, newborn, and child health. In this context, the Japanese government launched the Ghana Ensure Mothers and Babies Regular Access to Care (EMBRACE) Implementation Research Project in collaboration with the Ghanaian government. This study aims to evaluate the implementation process and effects of an intervention to increase the continuum of care for maternal, newborn, and child health status in Ghana.

**Methods/Design:**

We will conduct a cluster randomized controlled trial using an effectiveness-implementation hybrid design in Dodowa, Kintampo, and Navrongo, Ghana. We will provide an intervention package to women living in randomly allocated intervention clusters. The study population is women of reproductive age between the ages of 15 and 49 years. The package includes: 1) use of a new continuum of care card, 2) continuum of care orientation for health workers, 3) 24-hour health facility retention of mothers and newborns after delivery, and 4) postnatal care by home visits. We will measure maternal, newborn, and child health outcomes for both intervention and implementation impacts. The intervention outcomes are continuum of care completion rate, rate of postnatal care within 48 hours, complication rate requiring mothers' and newborns' hospitalizations, and perinatal and neonatal mortality. The implementation outcomes are intervention coverage of the target population, intervention adoption and fidelity, implementation cost, and sustainability.

**Discussion:**

In this trial, we will investigate how successful continuum of care can contribute to improving maternal, newborn, and child health outcomes. If successful, this model will then be implemented further in Ghana and other neighboring countries.

**Trial registration:**

Current Controlled Trials ISRCTN90618993. Registered on 3 September 2014.

## Background

The United Nations’ Millennium Development Goals (MDGs) 4 and 5 seek to improve maternal, newborn, and child health (MNCH) status. Their progress, however, has been minimal [1-4] and uneven across countries, especially in Sub-Saharan Africa [5-8]. Unless drastic improvement is attained, most countries in Sub-Saharan Africa will not meet MDGs 4 and 5 [5].

In this context, global health agencies have advocated continuum of care (CoC) as a new paradigm to overcome MNCH challenges [9,10]. The term CoC is well known in nursing for palliative [11,12] and mental health care [13]. In the public health area, the concept of CoC has been used in HIV and AIDS care [12,14], linking HIV diagnosis, care initiation, therapy retention, and low viral load maintenance. However, for MNCH, the definition of CoC has not yet been clearly established, although several attempts have been made [11,15-17]. So far, CoC has been explained by sequential time and space dimensions of MNCH activities. The time dimension lasts from pre-pregnancy for women through childhood. The space dimension links homes and communities through health facilities [17].

The CoC in MNCH provided a framework to improve health care for both women and their children by implementing integrated interventions [11,18]. Such intervention packages addressed different time stages in the CoC [19-21]. Additionally, single interventions were also efficacious for both women and their children [18]. Examples include newborn care preparedness by community-based approaches [22,23], use of insecticide-treated nets in pregnancy [24], and iron-folic acid supplementation [25,26]. Furthermore, financially focused strategies attempted to increase the demand for MNCH health services [27-30].

In the space dimension, the emphasized linkage is between the home and the first-level facility or the hospital. In most African countries, newborns are vulnerable to care delays and many newborn deaths occur at home [31]. Thus, interventions provided appropriate care at home, strengthened health system supports, and improved household and community practices and actions [32]. For both women and children, positive impacts were identified by training traditional birth attendants [33] and increasing emergency referral systems in the community [34]. Although these interventions improved MNCH outcomes, long-term sustainability remains a challenge [35], and it is unclear whether they were effective in ‘real-world’ settings. To make the best use of the available interventions, we need to know how they work in such settings [36].

Moreover, it is unclear whether health outcomes will be improved by filling the gaps in the CoC. A study estimated that neonatal mortality would be reduced by between 36 and 67% if all standard MNCH care packages (such as the antenatal care (ANC) package, skilled maternal and immediate newborn care package, and emergency obstetric care package) covered 90% of pregnant women [37,38]. However, evidence-based studies are scarce that demonstrate the effectiveness of improved CoC on health outcomes by addressing these gaps.

In 2010, the Japanese government presented new global health initiatives at the United Nations High-Level Plenary Meeting on MDGs [39]. The statement expressed a strategy to accelerate Japan’s concerted efforts to help achieve health-related MDGs in developing countries, particularly for maternal and child health [40]. The initiative comprised a model program, the Ghana Ensure Mothers and Babies Regular Access to Care (EMBRACE) program. This program exploits a package of effective interventions to improve the health of mothers and children through the CoC approach. A special feature of the initiative was the Japanese government’s clear intention to implement an ‘evidence-based intervention’ [41]. To extend the benefits of the Ghana EMBRACE initiative and evidence-based practices in health policy, the Ghana EMBRACE Implementation Research Project was launched in 2012 [42,43]. The project team comprised researchers from the University of Tokyo and the Ghana Health Service (GHS), including Dodowa, Kintampo, and Navrongo Health Research Centres (HRC). Based on formative research results, the project team developed interventions and will implement them in three Health and Demographic Surveillance System (HDSS) sites in Ghana.

Ghana is one of the countries that face a significant challenge to improving MNCH status [8,44-46]. Although it has made progress in different HDSS sites [47-52], according to the currently available evidence, Ghana is not on track to meet these MDGs [53]. In particular, MNCH status is poor in remote areas [47,49], and care-seeking decisions are delayed for ill mothers and children [54,55]. Neonatal death is associated with maternal factors such as multiple gestations and inadequate birth spacing [56]. Additionally, mothers and children do not use all MNCH services continuously. In particular, mothers pay less attention to newborn care. In almost half of home deliveries, postnatal care (PNC) was not completed [45]. Approximately 40% of mothers who delivered at home reported that they received PNC for themselves, while only 16% reported receiving it for their newborns [57]. However, when mothers appropriately used the antenatal, delivery, and postnatal health services, the risk of neonatal death was reduced [56].

MNCH is often influenced by a complex interaction of economic, financial, social, cultural, and clinical factors. Ghana has multiple localities formed by the particular characteristics of each area. Poor infrastructure often limits access to emergency care [55]. In certain areas, a traditional illness, *asram*, could be one cause of care-seeking delay for ill children [58]. The diversity of local characteristics implies the need for flexibility in health service provision [59,60]. This explains why implementation trials should be conducted in Ghana in different actual settings.

This study proposes specific objectives for the intervention and implementation phases. The intervention objective is to evaluate the impact of increased CoC completion on MNCH status in Ghana. The implementation objective is to evaluate intervention acceptability in different settings in Ghana.

In light of pragmatic situations in the intervention settings, we included the following as CoC components:

1. ANC delivered at least four times by MNCH health service providers at a health facility, in the community, or at home;
2. Delivery assisted by skilled birth attendants (SBAs); and
3. PNC delivered three times by MNCH health service providers at a health facility, in the community, or at home, within 48 hours, at seven days, and at six weeks postpartum.

## Methods/Design

### Study design

We will conduct a cluster randomized controlled trial using an effectiveness-implementation hybrid design [36,61,62]. In this study design, we will assess the effects of both an intervention package and its implementation process. An effectiveness-implementation hybrid trial is an innovative design to shorten the time from intervention development through its implementation in a real-world setting [63,64]. This study design is categorized into three types. The type one design focuses on testing the intervention while observing or gathering information on the implementation. The type two design simultaneously tests the intervention and its implementation strategy. The type three design focuses more on testing implementation strategy while observing or gathering information on intervention outcomes [61]. In our study, we will adopt the type two hybrid design to determine both the impact of the intervention on CoC completion in MNCH and the acceptability of the implementation strategy by GHS.

This design will assess the effectiveness of intervention and implementation by using cluster randomized allocation to divide participants into intervention and control arms. For trial sustainability and scalability, we will consider site-specific contexts in designing interventions [59]. We will conduct the study in three stages: baseline, implementation, and follow-up phases. Acceptability of the trials will also be evaluated through monitoring ongoing trials.

### Study site

**Figure 1:**
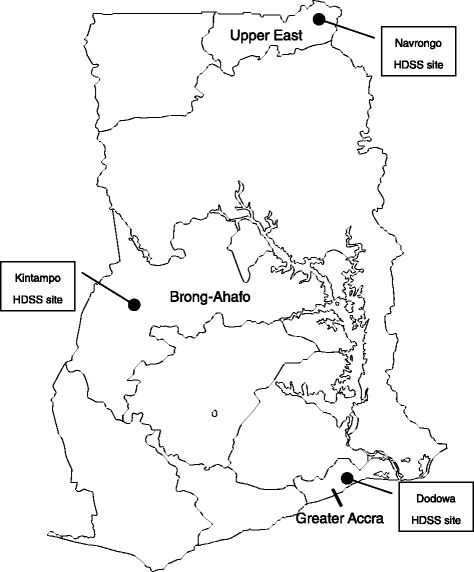
**Map of health and demographic surveillance system sites.** Ghana has health and demographic surveillance system (HDSS) sites in Dodowa, Kintampo, and Navrongo. The HDSS areas involve highly reliable semi-annual recording of vital demographic events occurring in residents of all households.

We will conduct the study at three different sites in Ghana; Dodowa (Greater Accra region), Kintampo (Brong-Ahafo region), and Navrongo (Upper East region), where the GHS runs HDSS sites (Figure 1). These three sites were selected because highly reliable HDSS data are available. HDSS involves semi-annual recording of vital demographic events occurring among residents of all households in the HDSS area: pregnancies, births, deaths, and migration. Other data are also collected and updated regularly, such as economic status, morbidity, and vaccination.

At the Dodowa HDSS site, the population was approximately 115,000 in 2011. It is located about 40 kilometers away from Accra [65]. Consequently, pregnant women from this site often prefer to deliver at health facilities in Accra. Service deliveries are challenging since the site land size covers about 40.5% of the Greater Accra region. Most of the communities are scattered, containing small populations [65,66].

At the Kintampo HDSS site, the surveillance population was approximately 200,000 in 2011. This site is a multi-ethnic area and farming is the most important economic activity. Apart from the central area, most villages are not supplied with electricity and are reached by dirt roads. Access to health facilities is a challenge in Kintampo [65,67], and a home delivery is often the first choice [68].

At the Navrongo HDSS site, the surveillance population was approximately 153,000 in 2011 [65,69]. Navrongo is the first area where the Community-based Health Planning and Services (CHPS) program was launched in Ghana. In that context, Community Health Officers (CHOs) have contributed to improving health status in communities. Among the three HDSS sites, only Navrongo is on track to achieve MDG 5 [48].

As the MNCH services are national priority areas in Ghana, various MNCH interventions have been implemented in the three HDSS sites. Representative interventions include Mobile Technology for Community Health (MoTeCH, between 2009 and 2012) [70], Quality of prenatal and maternal care (Qualmat, between 2009 and 2014) [71], and Ghana Essential Health Interventions Programme (GEHIP, between 2009 and 2014) [72] in Navrongo; early neonatal vitamin A supplementation in improving child survival (Neovita, between 2010 and 2012) [73], and Newborn home intervention study project (Newhints, between 2008 and 2010) [74] in Kintampo; and the conditional cash transfer project (GLST, between 2009 and 2014) [75] and Neonatal Quality Improvement Programme (NQIP, between 2011 and 2012) in Dodowa.

### Randomization and allocation

**Figure 2:**
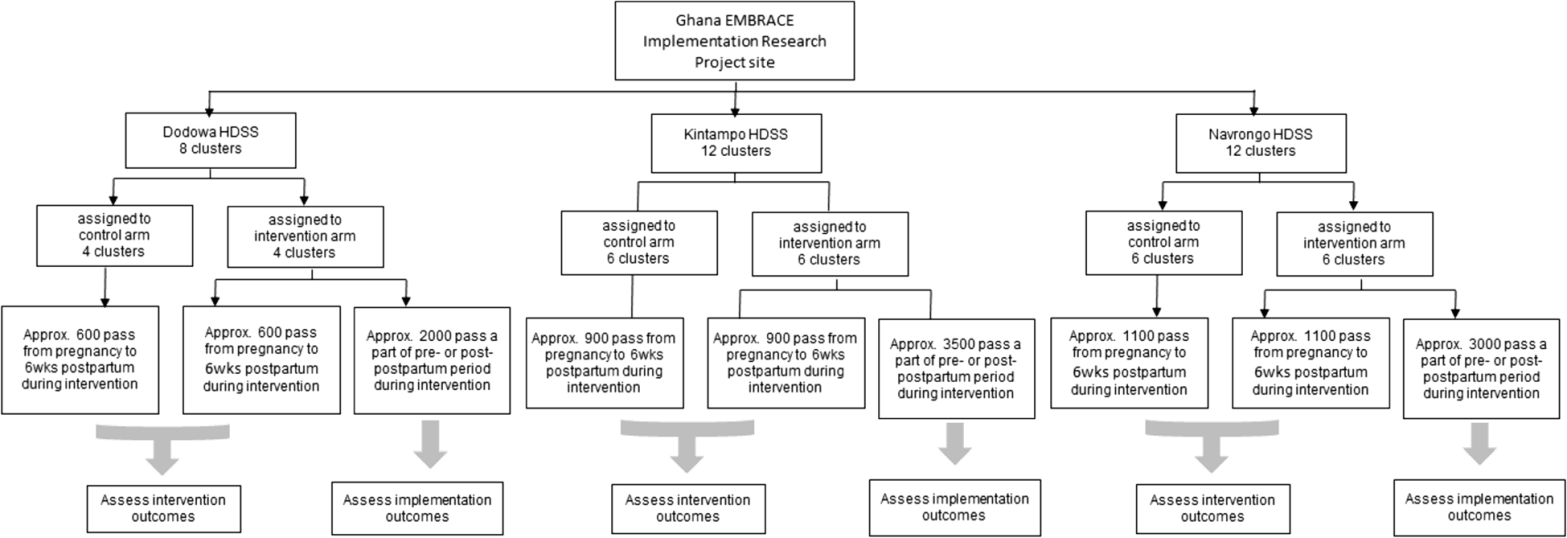
**Survey flow diagram for participant recruitment and inclusion.** Sub-districts were defined as a cluster unit. In total, 32 clusters were chosen as the study targets; eight clusters in Dodowa, 12 clusters in Kintampo, and 12 clusters in Navrongo. Half were allocated to the intervention arm. HDSS; Health and Demographic Surveillance System.

Each HDSS site contains two districts, and the districts comprise multiple sub-districts, which are the minimum health administration units in Ghana. We defined the sub-district as a cluster unit. Only Jema and Dumso (which used to be a part of Jema) sub-districts in the Kintampo site were combined to form a cluster. In the three HDSS sites, a total of 36 sub-districts were included. Of them, we excluded four sub-districts of Navrongo where another MNCH project is planned. In total, we chose 32 clusters for our study targets (Figure 2): eight clusters in Dodowa, 12 clusters in Kintampo, and 12 clusters in Navrongo. We allocated half of the sub-districts to the intervention arm and the other half to the control arm. At least one hospital was allocated to the intervention arm in each site, since the majority of facility deliveries are done in hospitals (Table 1). Due to the nature of the intervention, masking was not feasible.

**Table 1:** **Number of facilities (As of June 2014)**

**HDSS site**	**Arm**	**Hospital**	**Health center**	**Functional CHPS zone**	**Private**
Dodowa	Intervention	1	1	13	5
	Control	0	3	21	4
Kintampo	Intervention	1	2	19	5
	Control	1	4	22	0
Navrongo	Intervention	1	3	18	4
	Control	0	3	18	0

CHPS, Community-based Health Planning and Services; HDSS, Health and Demographic Surveillance System.

We matched the clusters within the site before randomization in the following aspects: population, number of deliveries in each cluster, and number of midwives available. Cluster randomization was preferred over individual-level randomization to minimize contamination, and also for the pragmatic purposes of a future scale-up of the intervention. The clusters were randomized by a data analyst, who was not a primary member of the study team, using computer-generated random sequences.

### Study population and selection criteria

Our study population is women of reproductive age between the ages of 15 and 49 years who live in the areas covered by the Dodowa, Kintampo, and Navrongo HDSS sites. Participants will be women who have given birth in the study area between 1 September 2012 and 30 June 2014 for the baseline period, and between 1 October 2014 and 30 September 2015 for the trial period. Exclusion criteria for the trials are women who refuse to participate in the intervention. We expect only a few women will meet it as the interventions will be implemented within the current service delivery system. In other words, they will receive the interventions unless they refuse national standard MNCH services. Exclusion criteria for the impact evaluations are those who decline to be interviewed or who have migrated out of the HDSS sites. We will also involve health service providers in the intervention to provide CoC services and to educate participant women in the CoC concept.

### Recruitment and consent of participants

We will deliver the interventions to all eligible women in the intervention arm. MNCH service providers will approach them when receiving MNCH care at health facilities or home. The expected recruitment period will be from August to September 2014 for the baseline survey, from October 2014 to September 2015 for the intervention, and from October to November 2015 for the follow-up survey. We will request informed consent form signatures from the survey participants. Additionally, we will obtain participants’ oral consent at their intervention entry point. Their participation will be voluntary and they are free to join or withdraw at any time.

### The Ghana EMBRACE interventions

#### Development of an intervention package

We developed a package of interventions based on a formative research survey conducted in three different HDSS sites (Table 2). In the survey, we identified the gaps, barriers, and promoters of service reception in continuous MNCH care. To address the actual MNCH conditions in Ghana and site-specific cultural differences in delivery location, geographic conditions, and health system capacity, we developed the following four interventions: the utilization of the CoC card (A-1), CoC orientation (A-2), 24-hour health facility retention of mothers and newborns after delivery (B-1), and PNC by home visits (B-2). We will implement A-1, A-2, and B-2 interventions in all three HDSS sites. However, B-1 will be implemented only in Dodowa and Navrongo because the number of midwives is not enough in Kintampo.

**Table 2:** **Interventions of Ghana EMBRACE implementation research project**

**Interventions**	**Implementations**	**Implementers**	**Targets**
(A-1) Utilization of CoC card	Encourage mothers to receive four ANC visits, facility delivery, PNC within 48 hours, at 7 days, and at 6 weeks	Health service providers who routinely provide MNCH at health facility, community, or home: doctor, midwife, nurse, CHO, and CHN	Women in prepartum/postpartum period who come to the health facility located in the intervention arm for ANC, delivery, and PNC
(A-2) Orientation of health service providers	Reorient supervisors in understanding CoC	Master trainers from GHS/EMBRACE researchers	Regional Health Administrations, DHMT, and SDHMT
	Reorient health service providers in understanding CoC	Trainer from DHMT/SDHMT who attended the training of trainers	Primary maternal and child health service providers of the health facilities in the intervention sites: midwife, CHO, CHN, doctor, nurse, and health assistant.
(B-1) 24-hour retention of mother and their newborns after delivery	Provide PNC within 48 hours by retaining mothers with newborns for at least 24-hours postpartum	Health service providers working at district hospital, health center, CHPS compound, or private clinic with midwife	Mothers who delivered at a relevant health facility and their newborns
(B-2) PNC by home visits	Provide PNC within 48 hours by visiting the home of mothers	Health service providers working at health center or CHPS compound	Mothers who gave birth but did not stay for 24 hours after delivery
			Mothers who delivered at home and their newborns

ANC, antenatal care; CHN, Community Health Nurse; CHO, Community Health Officer; CHPS, Community-based Health Planning and Service; CoC, Continuum of Care; DHMT, District Health Management Team; SDHMT, Sub-District Health Management Team; GHS, Ghana Health Service; MNCH, maternal, newborn, and child health; PNC, postnatal care.

In the control arm, Ghanaian government will provide conventional MNCH services. In most of the primary level health facilities, basic MNCH services will be provided. The delivery and laboratory services are provided mainly in the secondary and tertiary level health facilities. ANC services include general check-ups for mothers, urine tests, hemoglobin tests, prevention of mother-to-child transmission (PMTCT) of HIV, nutritional support, tetanus toxoid immunization, and health education for birth preparedness and maternal complications. Delivery services include skilled delivery, facility referral, and emergency obstetric care. PNC services include general check-ups for mothers and children, body weights, infant vaccinations, hemoglobin tests, nutritional support, health education for breastfeeding and child care, and family planning counselling. Home visit check-ups are also provided, however, home visit PNC within 48 hours is poorly adopted.

#### A-1 Utilization of the continuum of care card

In addition to the maternal health record book, health service providers will provide the MNCH CoC card to all women who receive ANC, delivery, and PNC assisted by SBAs (Table 3). When these women receive follow-up services, health service providers place a sticker on the card to show that they complied with the CoC; this includes four ANC visits, delivery assisted by SBAs, and three PNC visits (within 48 hours, at seven days, and at six weeks after delivery). Additionally, health service providers record on the CoC card: provision of essential services and health education; blood tests for assessing hemoglobin, blood group, and Rhesus factor; intermittent preventive treatment (IPT) for malaria; tetanus toxoid immunization; early initiating and exclusive breastfeeding; family planning; preparing items for delivery and baby; arrangement of transportation for delivery, caregiver, and calling health service providers after delivery; and the presence of danger signs during pregnancy and after delivery for mother and newborn.

**Table 3:** **Maternal, newborn, and child health continuum of care card contents**

**Contents**	**Main components**
CoC services	Four ANC visits
	Delivery with skilled birth attendant
	Three PNC visits
Essential services	Hemoglobin test
	Malaria drug (IPT)
	Tetanus toxoid immunization
	Blood group and Rhesus factor
Health education	Items for delivery and baby
	Caregiver
	Transportation for delivery
	Call a health service provider after delivery
	Early initiate and exclusive breast feeding
	Family planning counselling
Danger signs	Identification of danger signs during pregnancy
	Identification of danger signs at delivery and after delivery for mother
	Identification of danger signs at birth and after birth for newborn

ANC, antenatal care; CoC, Continuum of Care; IPT, Intermittent Preventive Treatment; PNC, postnatal care.

#### A-2 Continuum of care orientation

We will implement the MNCH CoC orientation in two stages. First, we will complete the training of trainers for supervisors of District Health Management Teams (DHMT) and Sub-District Health Management Teams (SDHMT). The trained supervisors will then conduct orientations for health service providers at hospitals, health centers, CHPS, and private clinics. We will focus mainly on introducing MNCH CoC concepts, their importance, the extent to which the MNCH CoC card could be used, and the protocols of other interventions.

#### B-1 24-hour health facility retention of mothers and newborns after delivery

In this intervention, we will encourage mothers to stay with their newborns at health facilities after delivery for at least 24 hours for PNC. This intervention targets only the health facilities where SBAs provide delivery services. In this intervention, mothers and newborns will receive necessary care in a health facility during the 24 hours after delivery. During their stay, we will provide them nutritious drink supplements (for example, Milo (Nestlé S.A., Vevey, Switzerland)). After ensuring all of the necessary health check points, health service providers will discharge them if neither mothers nor babies show any danger signs.

#### B-2 Postnatal care by home visits

We will encourage CHOs to visit mothers and newborns for PNC within 48 hours after delivery. This intervention is composed of two steps. The first step is delivery notification; when home delivery occurs, community health volunteers, community key informants, or traditional birth attendants will inform the CHOs that the labor or delivery occurred in the community. The second step is PNC by home visits; CHOs visit mothers and newborns for PNC within 48 hours postpartum.

#### Procurement

Where necessary, we will provide beds for postpartum rest, rechargeable lamps or solar lanterns, torchlights for all B-1 intervention facilities, and motorbikes for the B-2 intervention facilities. We will also provide care materials for both eligible categories of health facility such as blood pressure apparatus for mothers and children, stethoscopes, thermometers, and pen lights.

#### Supportive activities

We will organize three types of supportive activities before the start of the intervention to implement the interventions smoothly into the communities. They include stakeholder meetings, community leader meetings, and community durbars. We will organize the stakeholder meetings in both the intervention and control arms. To avoid contamination of the intervention effect, the community leader meetings and community durbars will be held only in the intervention arm.

#### Monitoring

In each HRC, we will form an intervention monitoring team. This team is responsible for monthly monitoring of all implementation activities of health service providers and for supervision of the DHMT/SDHMT supervisors. The EMBRACE researchers will meet monthly to discuss the issues raised during the monitoring and provide feedback to the monitoring team and the DHMT/SDHMT supervisors.

### Data collection

We will assess the impact of the intervention by interview survey using a semi-structured questionnaire and HDSS data. We will also assess the acceptability of the implementation process by monthly monitoring and supervision, and by conducting a survey of eligible women at the HDSS sites. Data managers of the HRCs will review all of the collected data for accuracy and completion. Data will be entered into computers twice, using Visual FoxPro software (Microsoft, Washington, United States). Verification checks will be done to correct any discrepancies in records.

#### Evaluation of intervention efficacy

We will assess the intervention efficacy using both survey and HDSS data, as described below:

1. By survey: to assess the intervention impact, we will conduct the interview survey at baseline and follow-up periods for sampled eligible women. For each survey, we will recruit approximately 500 women from each HDSS site (in total, 1,500 women for each survey). We will select them through the HRC pregnancy registers using the following two steps: (a) creating geographical units (GU) in proportion with the population size of the cluster and selecting 50 GUs from each HDSS site, and (b) selecting 10 eligible women from each GU. We will select the GUs and women using computer-generated random numbers.
  The questionnaire items are: sociodemographic and socioeconomic characteristics; MNCH services uptake; health complications during pregnancy, at delivery, and during postnatal period; pregnancy outcomes; and care-seeking behaviors. To control for the effects of health care provider and health facility characteristics on the study outcome, we will conduct health care provider assessment and health facility assessment at the baseline survey for both intervention and control arms. The data we will collect includes information on service provision, knowledge of CoC, supervision, community support, job satisfaction at health care provider level, infrastructure, human resources, MNCH service availability, annual statistics of key MNCH indicators, current practice of retaining mother and newborn for the first 24 hours, and provision of PNC by home visit within 48 hours postpartum.
  Prior to the survey, we will complete training for research assistants at each site. The EMBRACE researchers will develop the survey questionnaire through several workshops based on the formative research questionnaire. We will develop the questionnaire in English and the research assistants will interpret it orally when they conduct interviews with participants. As the local languages are not always written, we will not use translated questionnaires. The trained research assistants will pre-test the questionnaire with 20 eligible women and the researchers will revise it accordingly.
2. By HDSS data: we will also obtain the HDSS data to assess the perinatal mortality rate (PMR) and the neonatal mortality rate (NMR) of HDSS sites. We will officially request that HRCs share the relevant data with the Ghana EMBRACE Implementation Research Project.

#### Evaluation of implementation process acceptability

We will assess the acceptability of the implementation process by monitoring, supervision, key informant interviews, and surveys, as described below:

1. By monitoring: to assess intervention adoption, we will collect data about service provision through monthly monitoring. Data sources will be the health facility register book and District Health Information Management System (DHIMS) reports. The EMBRACE researchers developed the monitoring items through several workshops.
2. By supervision: to assess intervention fidelity, we will collect monthly supervision data to check the consistency of intervention implementation.
3. By key informant interviews: we will conduct key informant interviews among DHMT and SDHMT supervisors, health service providers, and mothers in the intervention arm.
4. By survey: we will ask about health education and essential services provided to the women in the baseline and follow-up surveys. We will ask women in the intervention arm about service preferences through the follow-up survey.

### Outcome measures

#### Intervention outcome measures

**Figure 3:**
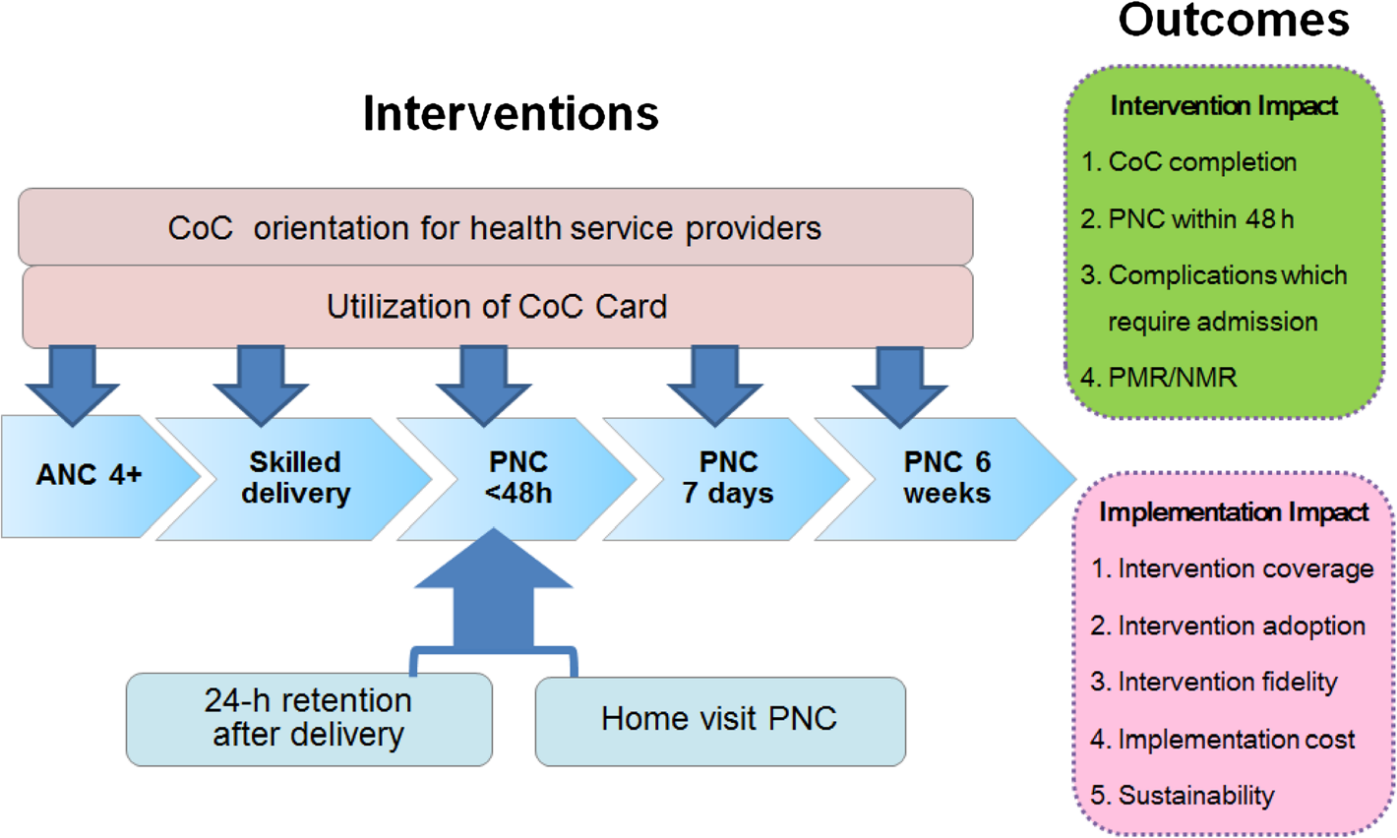
**Conceptual framework.** The intervention package includes the utilization of the CoC card, CoC orientation for health service providers, 24-hour health facility retention of mothers and newborns after delivery, and home visit PNC. The outcomes will be measured for both intervention and implementation aspects (ANC, antenatal care; CoC, Continuum of Care; NMR, neonatal mortality rate; PMR, perinatal mortality rate; PNC, postnatal care).

The primary outcome of the intervention is CoC completion rate of mothers and their children (Figure 3 and Table 4). The secondary outcomes include the PNC rate within 48 hours, the complication rate requiring mothers’ and newborns' hospitalizations, and the PMR and NMR. The PMR is defined as fetal deaths during any period of pregnancy and newborn deaths within seven completed days after birth. The NMR includes early neonatal deaths occurring during the first seven days of life and late neonatal deaths occurring after seven days but before 28 completed days of life [76,77].

**Table 4:** **Outcomes of Ghana EMBRACE implementation research project**

**Study objective**	**Outcome variables**		**Methods**	**Data source**
**Intervention outcomes**				
To examine the effect of the EMBRACE interventions on CoC completion	1) CoC completion rate	Rate of mothers and their newborns who completed CoC: ANC 4 visits, delivery assisted by SBAs, PNC 3 times (within 48 hours, at 7 days, at 6 weeks postpartum)	Quantitative analysis	Interview using semi-structured questionnaire
				Monitoring data
	2) Rate of PNC within 48 hours	PNC rate of mothers and newborns within 48 hours postpartum by 24 hours retention at health facility or home visit	Quantitative analysis	Interview using semi-structured questionnaire
				Monitoring data
To evaluate the impact of the interventions on MNCH status	3) Complication rate of mothers and newborns	Complication rates which require mothers’ and newborns’ hospitalizations for more than 24 hours	Quantitative analysis	Interview using semi-structured questionnaire
	4) PMR and NMR	PMR: fetal deaths during any period of pregnancy and newborn death within 7 completed days after birth. Early NMR: neonatal deaths occurring during the first 7 days of life. Late NMR: neonatal deaths occurring after 7 days but before 28 completed days of life	Quantitative analysis	HDSS data
				Interview using semi-structured questionnaire
**Implementation outcomes**				
To evaluate the acceptability of the interventions in different settings in Ghana	1) Intervention coverage	Percentage of women covered by intervention	Quantitative analysis	Interview using semi-structured questionnaire
				Monitoring data
	2) Adoption	Percentage and types of settings and staff adopted in the intervention	Quantitative analysis	Monitoring data
			Qualitative summary of key informant interviews	Supervision data
				Notes from key informant interview
	3) Fidelity	Adherence to the protocol and quality of intervention delivery	Quantitative analysis	Monitoring data
			Qualitative summary of key informant interviews Monthly meeting of project coordinators	Supervision data
				Notes from key informant interview
				Discussion notes of coordinators’ meetings
	4) Implementation cost	Direct measures of implementation cost and additional expense of implementation costs	Quantitative summary	Costing format
	5) Sustainability	Institutionalization of interventions or practice Passage, cycle or routine, and niche saturation	Discussions incorporating results of intervention research with project supervisors	Discussion notes of project meetings including district health officer
				Notes from key informant interview with health workers and district health officers
			Qualitative summary of key informant interviews	

ANC, antenatal care; CoC, Continuum of Care; HDSS, Health and Demographic Surveillance System; MNCH, maternal, newborn, and child health; NMR, neonatal mortality rate; PMR, perinatal mortality rate; PNC, postnatal care; SBA, skilled birth attendant.

#### Implementation outcome measures

The implementation outcomes will be measured by five outcomes: 1) intervention coverage of target population, 2) adoption and 3) fidelity in CoC card utilization or PNC within 48 hours by mothers’ retention at health facility or by home visit, 4) implementation cost, and 5) sustainability [64,78,79].

### Sample size

We made a calculation for two types of sample size to measure different outcomes. At first, to measure the CoC completion rate, we used the interview survey and calculated a total of 1,500 women for the sample size. Second, to measure the NMR, we used a HDSS data and calculated a total of 15,000 women for the sample size.

To calculate the sample size of the CoC completion rate, we used the data of the formative research collected in the previous year in the same sites. According to the data, the CoC completion rate was 8.0%. The coverage of four ANC visits, delivery attended by SBAs, PNC within 48 hours postpartum, and PNC at two weeks were 86.6%, 75.8%, 13.0%, and 60.0%, respectively. The lowest coverage was identified at PNC within 48 hours postpartum, and if this coverage increases to more than that of PNC at two weeks, the CoC completion rate would also increase to 60.0%. For that, we estimated that coverage of four ANC visits would be improved from 86.6 to 95.0%, and calculated the sample size using an intraclass correlation coefficient (ICC) of 0.02675 determined in the formative research. The ICC was estimated by considering the differences in the sizes of clusters using multilevel regression with a random intercept at the cluster level. The confidence interval (CI) was 95%, and power was 80%. Adding 10% for potential attrition, the total sample size was calculated at 1,500 for each baseline and follow-up survey period. Therefore, we estimated approximately a 500-person sample size for each HDSS site (1,500 in total).

In addition to the interview survey, we will also use the HDSS data to evaluate the effect of the interventions on perinatal and neonatal mortality. For the baseline and follow-up survey periods, HDSS data will capture 15,000 pregnancy cases, respectively. The sample size was estimated according to the following assumptions: a 25% reduction of PMR (from 31 to 23 per 1,000 pregnancies), with 95% CI, 80% power, 32 clusters, and an ICC of 0.0007256. The ICC was based on previous MNCH research conducted in the study area [74] and the sample size was calculated at 13,548. In addition, we added 10% for potential attrition.

### Statistical analysis

#### Continuum of care completion and morbidity

We will conduct baseline and follow-up interview surveys to assess changes in each outcome in both the intervention and control arms. To minimize overestimation of intervention impact, we will estimate all intervention impact outcomes with an intention-to-treat analysis. In this analysis, individuals’ outcome data are analyzed according to the allocated arm regardless of the place where they received care. Also, all eligible individuals are included in the analysis regardless of whether they provided outcome data [80].

We will conduct a descriptive analysis to assess the distribution of various factors related to the mothers and newborns under study. To evaluate the average effectiveness of interventions on CoC completion, we will apply the generalized estimating equations model with both continuous and binary outcomes [80]. This model uses data on all mothers, including those with incomplete data, over the period from 16 weeks of pregnancy to six weeks postpartum. Adjustment factors include basic demographic characteristics, socioeconomic characteristics, and facility characteristics. The data analysis will mostly be conducted using Stata version 13 (StataCorp LP, College Station, Texas, United States).

#### Mortality rate

PMR and NMR will be calculated based on the number of live births and perinatal deaths or neonatal deaths as a total of the three HDSS sites, as well as at each HDSS site. These outcomes will be compared before and after interventions.

#### Adoption of postnatal care within 48 hours and continuum of care card utilization

Implementation impact will be evaluated by means of descriptive statistical analysis. The qualitative data will be coded and categorized.

### Ethical considerations

#### Approval

Ethical approval was obtained from the Ethics Review Committee of the GHS (reference: GHS-ERC: 13/03/14), the Institutional Review Boards of Dodowa HRC (reference: FGS-DHRC: 280214), Kintampo HRC (reference: 2014–11), and Navrongo HRC (reference: NHRCIRB137) in Ghana, and from the Research Ethics Committee of the University of Tokyo in Japan (reference serial number: 10513).

#### Individual and community consent

Informed consent will be obtained from all survey participants before their inclusion in the study. We will record the consent through a signature or thumbprint. Participants will be withdrawn from the study if they experience a serious or intolerable adverse event, develop or disclose symptoms or conditions listed in the exclusion criteria, or require early discontinuation for any other reason. This will not affect their normal service delivery at any of the health facilities.

Permission for conducting the intervention study will be sought from the local health authorities and community leaders before initiating the study.

#### Benefits and risks

This intervention package is not invasive. Thus, the participants will not be exposed to marked risks. By participating in this study, they benefit by improved CoC knowledge and care of mothers and newborns. The MNCH service providers will receive a training session about CoC and the procedures to be performed. Health facilities of the intervention arm will receive a set of PNC services, motorbikes, or rest beds if they are not available ones. We will introduce the intervention to the control arm participants immediately when a positive impact is identified. We will encourage referral to a health facility if we identify minor, acute, or chronic illness in mothers or newborns in either the intervention or control arms.

#### Confidentiality of information

All information obtained through this study will be confidential. Access to information will be limited to research assistants for conducting interviews and data entry management staff. Study records will be identified only by means of study identification numbers.

### Dissemination of trial findings

The results of the study will be presented first to community members and their leaders in HDSS sites. In addition, policy briefs will be developed in collaboration with the Policy, Planning, Monitoring, and Evaluation Division of the GHS and submitted to the Office of the Director-General of GHS and the Family Health Division. Presentations will be made at the GHS Directors meeting, the Senior Managers Meeting, and international conferences.

Trial findings will also be disseminated in scientific meetings and papers on the intervention impact on improving CoC, the impact of increased CoC on MNCH status, the acceptability of the interventions, and the strategies for Japan's international health policy for MNCH.

## Discussion

This paper describes the protocol for a cluster randomized controlled trial to evaluate the impact of increased CoC completion on MNCH status and the acceptability of the interventions in different settings in Ghana. The interventions will be implemented in close consultation with local health administrative offices that have direct responsibility for supervising health facilities. Ongoing feedback will be provided through routine supervision. Additionally, we developed the study materials in close cooperation with policymakers in Ghana.

The study has limitations. First, the short intervention period may limit the power to fully measure the intervention and implementation impacts. The period is short because we needed repeated discussions to develop intervention packages aimed at a sustainable and scalable intervention design in a real-world setting. The project takes into account the next scale-up phase which will likely last years, during which the impacts could be measured more accurately. To reduce this limitation, we adopted the effectiveness-implementation hybrid trial that is an advantageous study design for time efficiency that enables rapid scaling of the intervention up to the national MNCH service standard. Second, our intervention impacts could be affected by previous or current projects implemented at the HDSS sites. To minimize it, we will control the potential effect for data analysis and carefully discuss the study findings.

## Trial status

The trial was registered in the International Standard Randomized Controlled Trial Number Register on 3 September 2014 (ISRCTN90618993). Recruitment for intervention commences in October 2014 and will continue until September 2015.

## Abbreviations

ANC: Antenatal care
CHO: Community Health Officer
CHPS: Community-based Health Planning and Services
CI: Confidence interval
CoC: Continuum of Care
EMBRACE: Ensure Mothers and Babies Regular Access to Care
GHS: Ghana Health Service
GU: Geographical Unit
HDSS: Health and Demographic Surveillance System
HRC: Health Research Centre
ICC: Intraclass correlation coefficient
MDGs: Millennium Development Goals
MNCH: Maternal, newborn and child health
NMR: Neonatal mortality rate
PMR: Perinatal mortality rate
PMTCT: Prevention of mother-to-child transmission
PNC: Postnatal care
SBA: Skilled birth attendant
